# The indirect association of subjective work performance with sleep quality and wellbeing among high-stress workers in Japan: a cross-sectional study

**DOI:** 10.3389/fpubh.2026.1822066

**Published:** 2026-07-23

**Authors:** Anna Kubota, Hisateru Tachimori, Akihiro Koreki, Yoshiaki Kanamori, Manae Uchibori, Shiyori Usune, Mitsuhiro Sado, Yasue Mitsukura, Akira Ninomiya, Ryutaro Shirahama, Akihiro Fujimoto, Kanako Inabe, Hiroaki Miyata, Masaru Mimura

**Affiliations:** 1 Department of Health Policy and Management, Keio University School of Medicine, Tokyo, Japan; 2 Endowed Course for Health System Innovation, Keio University School of Medicine, Tokyo, Japan; 3 Department of Neuropsychiatry, Keio University School of Medicine, Tokyo, Japan; 4 Mindfulness & Stress Research Center, Keio University, Tokyo, Japan; 5 Keio University Health Center, Tokyo, Japan; 6 Department of System Design Engineering, Faculty of Science and Technology, Keio University, Yokohama, Kanagawa, Japan; 7 Faculty of Science and Technology, Keio University, Yokohama, Kanagawa, Japan; 8 Medical Headquarters, Eisai Co., Ltd., Tokyo, Japan; 9 Center for Preventive Medicine, Keio University, Tokyo, Japan

**Keywords:** Japanese workers, mediation analysis, occupational health, presenteeism, sleep quality, subjective work performance

## Abstract

In today’s fast-paced and demanding working society, promoting wellbeing has become increasingly important. While sleep quality is widely recognized as a critical determinant of wellbeing, the specific pathways linking the two remain underexplored, particularly in high-stress occupational settings. Understanding these associations may enable the development of more effective strategies for promoting wellbeing in this population. Recently, work performance (presenteeism) has garnered attention for its potential intermediary role, as it may play a pivotal role in supporting employees’ wellbeing. At the same time, work performance itself is known to be closely related to sleep disturbances. However, the mediating effect has not been thoroughly examined. This study aimed to investigate whether presenteeism serves as a link in the association between sleep quality and wellbeing in a working population. A total of 129 high-stress office workers in Japan participated in this observational study. Sleep quality, presenteeism, and wellbeing were assessed using validated measures, and mediation analysis was conducted using the PROCESS macro with bootstrap samples to estimate indirect associations. Results revealed associations between sleep quality and presenteeism (*r* = − 0.306, *p* < 0.01), as well as between sleep quality and wellbeing (*r* = − 0.213, *p* < 0.01), indicating that poorer sleep (i.e., higher PSQI scores) was linked to both higher presenteeism (lower performance) and reduced wellbeing. Furthermore, presenteeism was found to statistically mediate the association between sleep quality and wellbeing. Even after adjusting for multiple covariates (age, sex, years of service, weekly working hours, occupational position, smoking status, and alcohol consumption), a significant indirect association was observed (Estimate = −0.446, 95% CI [−0.919, −0.072]). These findings suggest that the importance of work performance as a potential intermediary factor between sleep and wellbeing among high-stress workers. This study provides novel insights into the occupational mechanisms associated with sleep quality and wellbeing and highlights the importance of addressing presenteeism as a critical intervention target in occupational health.

## 1 Introduction

In today’s fast-paced and demanding working society, promoting wellbeing has become increasingly important. Among the fundamental factors associated with wellbeing, sleep quality has received considerable attention. Higher sleep quality enhances cognitive efficiency and emotional stability, which may contribute to greater life satisfaction (1, 2). Conversely, poor sleep quality is often accompanied by a cascade of adverse outcomes, including cognitive impairments, emotional instability, and heightened stress responses, which collectively may undermine both personal health and psychosocial functioning (1, 2).

In occupational contexts, a growing body of evidence suggests that reduced sleep quality is associated with negative work-related outcomes, such as diminished work engagement, lower subjective productivity, and increased presenteeism—being physically present at work but performing below one’s capacity (3, 4). While these effects are relevant across all workers, they are particularly pronounced among employees in psychologically demanding environments, where poor sleep quality and its consequences tend to be more severe (5). In such contexts, reduced sleep quality is closely related to impaired workplace performance, which in turn is thought to be associated with overall life satisfaction and psychological wellbeing.

In this study, presenteeism was used as a measure of subjective work performance, reflecting the degree to which health-related factors are associated with reduced workplace performance. This line of reasoning suggests a hypothetical model in which work performance (as measured by presenteeism) may serve as a key intermediary factor in the relationship between sleep quality and wellbeing. The significance of these interconnected associations is likely to be more salient among individuals working in high-stress environments, underscoring the need for empirical investigation.

Against this backdrop, integrated interventions that simultaneously target both sleep hygiene and occupational factors have recently gained attention. Notably, van Elk et al. (6) demonstrated that a multifaceted intervention addressing both sleep practices and occupational factors such as shift scheduling, was associated with improved sleep quality, reduced fatigue, and enhanced recovery needs among healthcare workers (7).

However, while these existing intervention and observational studies have established broad associations among work stress, sleep, and performance, several critical gaps remain in the literature, which limits their applicability to strategic occupational health.

First, most previous research has focused on broad, heterogeneous worker populations, leaving the specific occupational mechanisms within vulnerable, high-stress worker cohorts relatively underexplored. High-stress individuals are at a disproportionately higher risk for severe sleep disturbances, making them a critical target for investigation. Second, while the broad concept of “labor productivity” encompasses multidimensional and objective economic outputs, its evaluation in high-stress settings requires a more proximal focus on how health problems manifest daily at work—specifically through presenteeism (subjective work performance). In high-stress environments, workers frequently attend work while unwell, leading to a subtle but significant deterioration in performance that may not be immediately captured by macro-level productivity metrics. Furthermore, previous occupational studies have rarely extended the chain of these associations to include psychological wellbeing (life satisfaction) as the ultimate outcome, despite its profound implications for sustainable employment and long-term mental health. Investigating presenteeism as a specific behavioral mediator can uncover a more actionable pathway through which poor sleep translates into diminished life satisfaction. To uncover the novelty of this research, it is essential to clarify this intricate mechanism by examining a structured statistical pathway within a dedicated high-stress sample, while stringently controlling for key confounding variables such as length of service and working hours.

Building on these insights, the present study was designed with a primary objective.

to investigate whether subjective work performance (presenteeism) indirectly links the association between sleep quality and wellbeing. Based on the conceptual framework that sleep quality serves as a foundational resource for daily functioning and performance, we proposed the following specific hypotheses:

*Hypothesis 1 (H1)*: Poorer sleep quality is significantly associated with lower wellbeing among high-stress workers.

*Hypothesis 2 (H2)*: The association between sleep quality and wellbeing is statistically mediated by presenteeism (i.e., decreased subjective work performance).

By testing this focused mediator model, this study seeks to contribute to the existing literature by offering empirical evidence on the potential mediating role of work performance and proposing actionable insights for supporting both individual and organizational outcomes.

## 2 Methods

This study is a secondary analysis of the data obtained in “A survey for Japanese Office workers to Assess Stress Issues and Sleep problems (J-OASIS Study). The original J-OASIS Study employed a cross-sectional online survey design conducted among Japanese desk-based office workers. A summary of the research methodology of the J-OASIS Study and details of the information used in this study are described here. Further details of the J-OASIS Study are described elsewhere (7).

### 2.1 Ethical approval

This observational study, conducted prospectively at Keio University School of Medicine, adhered to the ethical principles outlined in the Declaration of Helsinki. The study protocol received approval from the Keio University School of Medicine Ethics Committee (Approval No. 2021-1031-4) and was registered in the University Hospital Medical Information Network (UMIN) clinical trials registry (UMIN000045528) under the title: “A survey for Japanese Office workers to Assess Stress Issues and Sleep problems (J-OASIS).” Prior to enrollment, written informed consent was obtained from all participants.

### 2.2 Participants

A total of 129 participants were included in the final analysis of this study. Participants were recruited through an online research platform using a convenience sampling strategy via an online recruitment platform (3H Medi Solution: https://global-3h.com/). To reduce variability in workplace environments, the study targeted office workers, with a particular emphasis on individuals experiencing occupational psychological stress. Participants were limited to those classified as high-stress workers based on our research objective of investigating sleep and work-related performance among populations at elevated risk for insomnia (8). Although this sample size (*N* = 129) is relatively small for complex multi-variable analyses, it represents a highly specific and stringently screened cohort of high-stress office workers.

Inclusion criteria were: (1) adults aged 20–65 years employed full-time; this specific age range was selected to represent the standard working-age population up to the conventional retirement age in Japan, and potential variances in stress levels across different life stages were statistically adjusted for by including age as a key covariate in the final models; (2) provision of written informed consent; (3) ability to attend scheduled research assessments; and (4) elevated levels of work-related stress. High-stress status was determined using the job demand-control model (9), following the methodology of our previous report (7). Based on the Brief Job Stress Questionnaire (BJSQ) (10), participants with scores exceeding the national mean for job demand (>2.14) and scores below the mean for job control (<2.53) were included (11). Demographic and occupational characteristics, including age, sex, years of service, weekly working hours, occupational position, smoking status, and alcohol consumption, were also collected at enrollment. Importantly, to account for the potential confounding effects of work experience and organizational familiarity on job stress, the length of service (years of service) was systematically included as a covariate in our statistical models.

Exclusion criteria encompassed: (1) employees on leave; (2) night shift workers (defined as working between 10 p.m. and 5 a.m.); (3) workers involved in physically intensive industries (e.g., manufacturing, construction, agriculture, food services); (4) individuals with current or past psychiatric diagnoses; (5) those taking psychotropic medications; (6) participants unable to consistently wear an Apple Watch for monitoring; and (7) principal investigators and research team members deemed ineligible to participate in the study.

### 2.3 Measurement tools

In J-OASIS study, various validated psychological and occupational scales were administered to capture multidimensional aspects of workers’ mental and behavioral health. For the present analysis, three core variables were examined: sleep quality, work performance, and subjective wellbeing. These were assessed using the following instruments:

#### 2.3.1 Pittsburgh sleep quality index (PSQI) (12)

The PSQI is a widely used and validated tool for assessing subjective sleep quality over the preceding month. It consists of 19 items across seven components, including sleep latency, duration, efficiency, and disturbances. In this study, as a representative measure of overall sleep perception, we utilized the specific item for “subjective sleep quality” (Question 6 of the PSQI). This item is scored on a 4-point Likert scale from 0 (very good) to 3 (very bad), where higher scores indicate poorer subjective sleep quality. This instrument is particularly effective in identifying sleep disturbances that may be associated with both mental health and workplace performance. The Japanese version of the PSQI has been previously validated and demonstrated high psychological reliability and validity in Japanese populations (12).

#### 2.3.2 WHO-health and work performance questionnaire short form (WHO-HPQ-SF) (13)

The WHO-HPQ-SF is a validated instrument designed to quantify subjective work performance deficits through absenteeism and presenteeism. For presenteeism items, lower scores indicate higher productivity loss, whereas higher scores on the absolute scale reflect better performance. In this study, we focused on presenteeism as a surrogate marker of work-related performance impairment, given its relevance in high-stress occupational contexts and its known association with sleep impairment.

Specifically, it was evaluated using the single specific item from the WHO-HPQ-SF asking: ‘On a scale of 0 to 10, how would you rate your overall job performance during the past 4 weeks?’ where 0 is the worst possible performance and 10 is the performance of a top worker. Following the methodology of a previous study (7), this “absolute presenteeism” score allows for the detection of performance fluctuations. The Japanese version of the WHO-HPQ-SF has been officially translated and psychometrically validated for occupational health research in Japan (13).

#### 2.3.3 Satisfaction with life scale (SWLS) (14)

The SWLS is a widely accepted measure for assessing an individual’s subjective evaluation of overall life satisfaction. This five-item scale, developed by Diener et al. (14), provides a global measure of perceived wellbeing. Respondents rate their agreement with statements on a 7-point Likert scale, ranging from 1 (“strongly disagree”) to 7 (“strongly agree”). The total score ranges from 5 to 35, with higher scores reflecting greater satisfaction with life. Its brevity and strong psychometric properties make it a valuable tool for examining wellbeing in relation to workplace and health variables. The psychometric properties of the Japanese version of the SWLS have been thoroughly evaluated, showing excellent internal consistency and construct validity.

#### 2.3.4 Use of other scales in the J-OASIS study

Although several additional scales were administered in the J-OASIS study—including the Brief Job Stress Questionnaire (BJSQ) (10), Job Content Questionnaire (JCQ) (11), Dutch Work Addiction Scale (DUWAS) (15), Epworth Sleepiness Scale (ESS) (16), Quick Inventory of Depressive Symptomatology (QIDS) (17), Multidimensional Assessment of Interoceptive Awareness (MAIA) (18), and Mindful Attention Awareness Scale (MAAS) (6)—they were not included in the present analysis, as our primary aim was to test a mediation model focused on sleep, productivity, and wellbeing.

#### 2.3.5 Covariates

To adjust for potential confounding factors, several demographic and occupational variables were included in the analysis. These included age, sex, and years of service. Weekly working hours were calculated by multiplying daily working hours by the number of working days per week. Additionally, occupational position (1 = management/middle management, 0 = general/contract staff), smoking status (1 = current smoker, 0 = non-smoker), and alcohol consumption (1 = regular drinker [≥2 times/week], 0 = occasional/non-drinker) were included as dichotomized dummy variables.

### 2.4 Statistical analysis

All statistical analyses were conducted using IBM SPSS Statistics (version 29). Descriptive statistics, including the demographic characteristics and scale scores, were summarized to evaluate associations among sleep quality, work performance, and wellbeing. To ensure data integrity, the scores for each scale were verified against their theoretical ranges: PSQI (item 6, 0–3), WHO-HPQ (0–10), and SWLS (5–35).

The hypothesized mediation model was tested using the PROCESS macro for SPSS (Model 4) developed by Hayes (19), which facilitates the estimation of both direct and indirect effects in mediation frameworks. Sleep quality, measured using item 6 of the PSQI, was entered as the independent variable (X), presenteeism (absolute presenteeism score from the WHO-HPQ-SF) as the mediator (M), and wellbeing, evaluated using the SWLS, as the dependent variable (Y). To ensure the robustness of the model and adjust for potential confounding effects, the following covariates were included in the analysis: age, sex, years of service, weekly working hours (calculated as daily working hours multiplied by working days per week). Additionally, several lifestyle and occupational factors were dichotomized as dummy variables: occupational position (1 = management/middle management, 0 = general/contract staff), smoking status (1 = current smoker, 0 = non-smoker), and alcohol consumption (1 = regular drinker [≥2 times/week], 0 = occasional/non-drinker).

The analysis evaluated the following statistical paths:

- *Path a (X → M)*: The association between sleep quality and work performance, hypothesizing that poor sleep quality is associated with lower performance.
- *Path b (M → Y)*: The association between work performance and wellbeing, while adjusting for sleep quality.
- *Path c’ (direct effect, X → Y)*: The direct association between sleep quality and wellbeing, independent of work performance.
- *Path c (total effect, X → Y)*: The combined direct and indirect associations.
- *Indirect effect (a × b):* The mediating role of presenteeism, calculated as the product of Path a and Path b.

The indirect association was tested using bias-corrected nonparametric bootstrap confidence intervals (95%) generated from 5,000 bootstrap samples. Significance was established if the confidence intervals did not contain zero. All *p*-values were two-tailed, with the significance level set at 0.05.

## 3 Results

### 3.1 Demographic and occupational characteristics of study participants

The mean age of participants was 41.65 years (SD = 10.81). The sex distribution included 61 males (47.3%) and 68 females (52.7%) Detailed characteristics, including years of service, weekly working hours, occupational position, and lifestyle factors, are summarized in Table 1.

**Table 1:** Demographic characteristics of study participants.

Characteristic	*N* or mean/median	% or SD
Basic demographics		
Age(years)	41.65	10.81
Gender		
Male	61	47.3
Female	68	52.7
Occupational characteristics		
Occupational position		
Management (manager/middle manager)	28	21.7
Non-management (general/contract)	101	78.3
Years of service	18.57	10.61
Weekly working hours	44.20	7.17
Lifestyle factors		
Smoking status		
Smoking	17	13.2
Non-smoking	112	86.8
Alcohol consumption		
Regular drinkers (≥2 times/week)	34	26.4
Occasional/non-drinkers (<2 times/week)	95	73.6
Main study variables		
PSQI “sleep quality” score	1.29	0.76
Presenteeism score	62.40	15.75
Wellbeing score	17.23	5.85

PSQI “sleep quality” score refers to the single-item measure of subjective sleep quality (range 0–3). Weekly working hours were calculated as (daily working hours × working days per week). Occupational position was categorized into management (including middle management) and non-management.

### 3.2 Associations among sleep quality, work performance, and wellbeing

Bivariate correlation analysis revealed significant associations among the key variables. Sleep quality was significantly associated with both presenteeism (work performance) (*r* = − 0.306, *p* < 0.01) and wellbeing (*r* = − 0.213, *p* = 0.015). Since higher PSQI scores reflect poorer sleep quality, these results indicate that poorer sleep is associated with lower work performance and reduced wellbeing. Additionally, work performance was positively correlated with wellbeing (*r* = 0.26, *p* < 0.01) (Table 2).

**Table 2:** Correlates of sleep quality, labor productivity, and wellbeing.

Variables	PSQI “sleep quality”	Presenteeism	(*N* = 129)
			Wellbeing
PSQI “sleep quality”	1.000		
Presenteeism	−0.306**	1.000	
Wellbeing	−0.213**	0.260**	1.000

Pearson’s correlation coefficients. **denote significance at the 1% level.

### 3.3 Mediation analysis of presenteeism in the association between sleep quality and wellbeing

**Figure 1:**
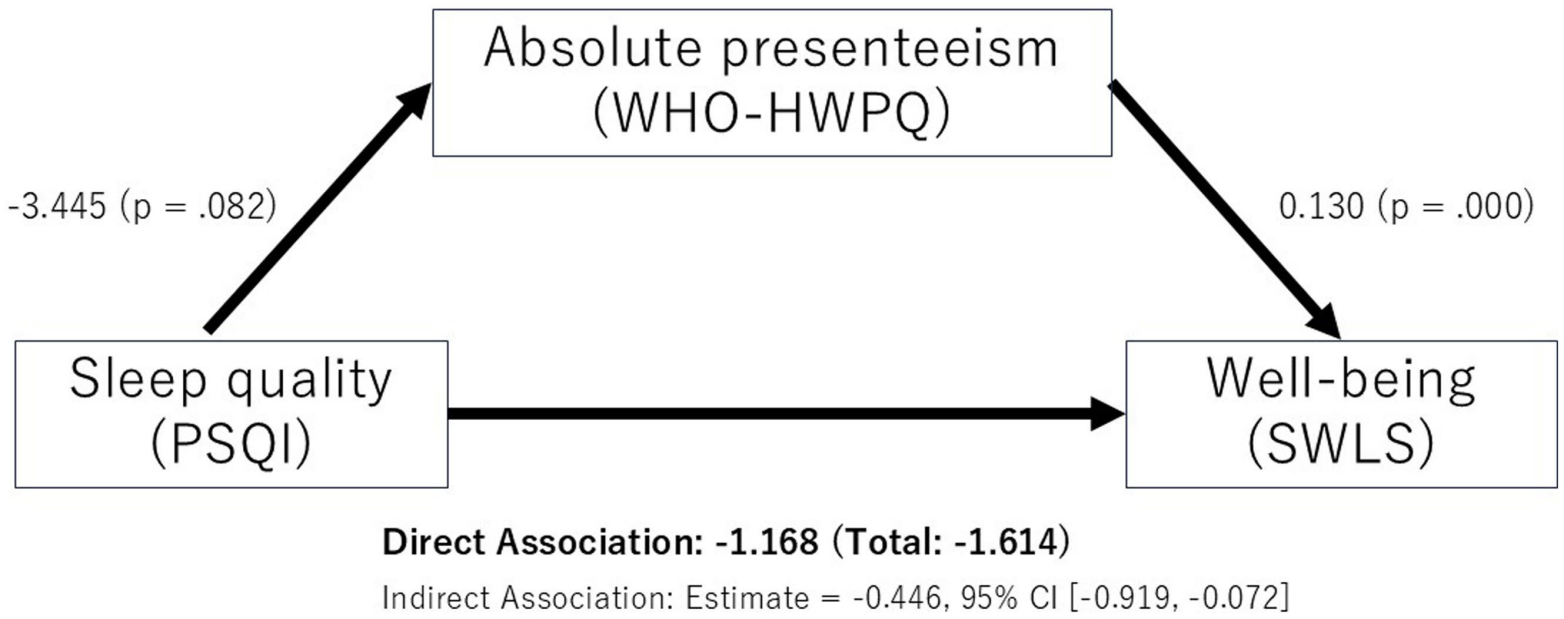
Adjusted for age, sex, years of service, weekly working hours, occupational position, smoking status, and alcohol consumption Values presented on the paths represent standardized regression coefficients (B) derived from the multivariate mediation analysis adjusted for age, sex, length of service, weekly working hours, occupational position, smoking status, and alcohol consumption, whereas Table 2 displays unadjusted Pearson's correlation coefficients.

The mediation model, adjusting for age, sex, years of service, weekly working hours, occupational position, smoking status, and alcohol consumption, is presented in Figure 1 and Table 3. The indirect association between sleep quality and wellbeing through presenteeism was statistically significant (Estimate = −0.446, 95% CI [−0.919, −0.072]), as the bootstrap confidence interval did not include zero. In contrast, the direct association of sleep quality with wellbeing, after controlling for presenteeism and the aforementioned covariates, was −1.168 (95% CI [−2.602, 0.267]), which did not reach statistical significance. These findings suggest that the statistical association between sleep quality and wellbeing is largely accounted for by its link with workplace performance (presenteeism), highlighting the potential intermediary role of work performance in this population.

**Table 3:** Mediating effects of labor productivity between sleep quality and wellbeing.

Effects	Effect value	Boot SE	Boot CI lower	Boot CI upper	(*N* = 129)
					Relative mediation effect
Total effect	−1.6135	0.7128	−3.0249	−0.2021	100.00%
Total indirect effect	−0.4460	0.2179	−0.9191	−0.0718	27.64%

## 4 Discussion

This study suggests that work performance (presenteeism) may play an intermediary role in the association between sleep quality and wellbeing among high stress workers, highlighting subjective work performance as a potential factor linking poor sleep to diminished life satisfaction. These findings suggest that the positive association between better sleep quality and enhanced wellbeing may be statistically accounted for, in part, by preserved workplace performance. This aligns with previous large-scale analyses showing that poor sleep is closely correlated with substantial productivity losses, which often co-occur with heightened stress and a lower overall quality of life (1, 20). Notably, our findings suggest that a meaningful portion of the association between sleep quality and wellbeing can be explained by presenteeism. While comparable statistical indirect associations have not been extensively reported in general working populations or clinical samples, this finding suggests that providing organizational support to mitigate presenteeism—such as task adjustments or flexible working conditions—may be beneficial for protecting the psychosocial wellbeing of workers experiencing poor sleep.

### 4.1 The role of subjective work performance as a mediator

Previous research has often identified presenteeism as a contributor to performance declines due to health issues (21). Although presenteeism overlaps with subjective productivity deficits, it specifically refers to being present at work while unwell, which may or may not capture all facets of objective performance. The current results suggest that subjective work performance—encompassing one’s perceived capacity to fulfill work tasks effectively—serves as an intermediary link through which sleep deficiencies are associated with reduced wellbeing. While prior studies have linked poor sleep to increased presenteeism (20), our findings extend these insights by demonstrating that poorer sleep quality, as assessed by higher PSQI scores, is significantly associated with lower absolute presenteeism scores on the WHO-HPQ, reflecting diminished subjective performance.

This finding underscores that work performance constitutes a major pathway associated with the link between sleep quality and life satisfaction. Given that work often forms a significant part of personal identity and fulfillment, diminished workplace performance may be associated with reduced overall wellbeing. Thus, maintaining performance levels may be pivotal not only for occupational success but also for sustaining broader psychosocial health.

### 4.2 Implications for policy and practice

The findings from this study underscore that sleep quality is significantly associated with both wellbeing and work performance, with presenteeism serving as a statistical mediator between sleep and overall wellbeing. Integrating sleep health into employee wellness programs could yield dual benefits—enhancing individual quality of life while also supporting workplace performance. Previous research has indicated that improvements in wellbeing may require a longer period to manifest following health interventions, whereas changes in work-related functioning such as subjective performance, can appear earlier (21). Our findings imply that monitoring changes in presenteeism could serve as an early, pragmatic marker of intervention effectiveness, enabling timely adjustments in occupational health programs. Building on this understanding, adding sleep-focused strategies to employee wellness initiatives could offer a practical and temporally sensitive means of promoting both individual and organizational outcomes. While our study did not specifically assess stress reduction strategies, the demonstrated role of work performance as a mediator underscores how programs that support healthier sleep schedules, mental health resources, and balanced workloads can jointly foster individual and organizational gains. Specifically, organizations could implement structured “sleep hygiene workshops” combined with flexible boundary management policies, such as introducing core working hours or cognitive behavioral therapy for insomnia (CBT-I) digital applications tailored for high-stress cohorts. Furthermore, establishing a workplace climate that psychologically permits employees to report health-related performance deficits without fear of negative personnel evaluations could facilitate early-stage managerial adjustments, such as temporary task redistribution, thereby preventing the escalation of presenteeism into severe wellbeing deterioration. Furthermore, implementing evaluation metrics that capture changes in these domains can enable organizations to better align their operational objectives with employee health outcomes, as supported by recent studies linking sleep improvements to enhanced subjective performance (22).

At a societal level, the economic burden associated with untreated sleep disorders is substantial, affecting both personal health and organizational efficiency (23). In light of this, policymakers are encouraged to develop comprehensive occupational health guidelines that integrate sleep interventions with regular assessments of both wellbeing and subjective work performance. Such measures, as evidenced by the literature, could effectively bridge the gap between individual health improvements and broader organizational benefits, thereby contributing to a more sustainable and healthy workforce (22, 23).

### 4.3 Limitations and future directions

This study has several important limitations that must be acknowledged.

First, its cross-sectional design precludes causal inferences.

While our analysis was based on a hypothesized directional model (Sleep → Work Performance → Wellbeing), a reverse causal pathway (e.g., lower wellbeing being associated with decreased performance, which in turn is linked to sleep disturbances) is statistically equivalent and highly plausible. Future longitudinal or interventional studies are strictly required to elucidate the temporal direction of these associations.

Second, all data were collected via online self-report surveys at a single time point, introducing a high risk of common method variance (CMV). Responses may have been influenced by participants’ subjective states or social desirability bias, potentially affecting the observed associations.

Third, the sample size (*N* = 129) is relatively small for detecting subtle direct effects, which limits the statistical power required for complex multi-variable models. Therefore, the lack of a statistically significant direct association (Path c’) must be interpreted with caution, and we cannot definitively claim “full mediation.” Nevertheless, it is important to note that this sample represents a highly targeted and stringently screened cohort from the J-OASIS study that strictly met the high-stress criteria. Within this specialized framework, our model allowed for a robust quantification of the prominent indirect link through which presenteeism connects sleep quality and wellbeing. Future studies with larger, multi-center longitudinal cohorts are warranted to comprehensively test the stability of this mediation structure.

Fourth, work performance was assessed using a single item for absolute presenteeism from the WHO-HPQ. As noted by the reviewers, a single subjective metric cannot fully capture the multidimensional nature of work performance and may be susceptible to daily mood fluctuations or recall bias. Incorporating objective metrics, such as actigraphy for sleep or quantifiable personnel evaluation data for productivity, is necessary for future research to validate these findings.

Finally, although we adjusted for several key occupational and lifestyle factors—including weekly working hours, years of service, occupational position, smoking, and alcohol consumption—other unmeasured confounding factors—such as exact income or specific job roles, were not fully addressed. However, the inclusion of these major covariates in our revised model enhances the robustness of the observed indirect association compared to a non-adjusted model. Despite these limitations, this study provides valuable preliminary insights into the occupational health of high-stress workers and highlights the importance of addressing presenteeism in targeted, longitudinal investigations.

## 5 Conclusion

Wellbeing among high-stressed workers is a complex construct; our findings suggest that subjective work performance (presenteeism) may serve as a significant statistical mediator in the association between sleep quality and wellbeing. This deepens our understanding of the occupational mechanisms linking sleep and life satisfaction, and underscores the importance of addressing sleep quality for such individuals. We believe that our findings will contribute to the development of comprehensive strategies to support high-stressed workers and identify innovative intervention targets in occupational health.

## Data Availability

The raw data supporting the conclusions of this article will be made available by the authors, without undue reservation.
